# Prenatal Screening for CMV Primary Infection: A Cost‐Utility Model

**DOI:** 10.1111/1471-0528.18080

**Published:** 2025-02-03

**Authors:** Gebrael El Hachem, Thomas G. Poder, Catherine Mc Carey, Soren Gantt, Fatima Kakkar, Marc Sab, Christian Renaud, Isabelle Boucoiran

**Affiliations:** ^1^ School of Public Health Université de Montréal Montreal Quebec Canada; ^2^ Women and Children's Infectious Diseases Center, CHU Sainte‐Justine Research Center Montreal Canada; ^3^ Research Center of the IUSMM CIUSSS de l'Est de l'Île de Montréal Montreal Canada; ^4^ Department of Obstetrics and Gynecology Université de Montréal Montreal Canada; ^5^ Department of Pediatrics Université de Montréal Montreal Canada; ^6^ Faculty of Medicine Université Catholique de Louvain Brussels Belgium

**Keywords:** cost utility, cytomegalovirus, prenatal screening

## Abstract

**Objective:**

Congenital cytomegalovirus (CMV) infection is a major cause of deafness and neurodevelopmental disability in children. Our objective was to assess the cost utility of first‐trimester serological CMV screening, compared to screening of high‐risk pregnancies and no serological screening.

**Design:**

A decision‐analytic model was created to compare the cost utility of three strategies from a healthcare sector perspective: universal first‐trimester serological screening, screening only of high‐risk pregnant women (both including antiviral prophylaxis in cases of primary infection) and serological testing triggered by foetal morphological ultrasound (no CMV serological screening).

**Setting:**

Canada.

**Population:**

Hypothetical population of 80 000 pregnant women.

**Methods:**

Probability, expected values and cost estimates were derived from published literature and local hospital and national insurance data.

**Main Outcome Measure:**

Cost per maternal and infant quality‐adjusted life year (QALY) lost.

**Results:**

Universal serological screening was superior to both screening of high‐risk women and no screening (utility of −0.42, −0.63 and − 0.87 QALY lost, respectively). Sensitivity analysis demonstrated that universal screening was the most cost‐effective strategy regardless of the incidence of primary infection, the acceptability of amniocentesis and the efficacy of antiviral prophylaxis. In the Monte Carlo analyses, universal serological screening was the most cost‐effective option in 96.36% of simulations. Universal serological screening would allow detection of 152 cases of primary maternal CMV infection and would prevent 29 cases of congenital CMV infection annually.

**Conclusion:**

Our findings support the adoption of a population‐based prenatal screening programme for the prevention of congenital CMV infection.

## 1 Introduction

Cytomegalovirus (CMV) is the leading cause of congenital infections [1]. It is a major cause of neurodevelopmental disorders, including sensorineural hearing loss [2, 3], visual impairment and/or cognitive delay [4], and is also associated with intrauterine foetal death, neonatal death and pregnancy termination [5, 6]. In Canada, as in most high‐resource settings, 1 in 200 neonates are congenitally infected with CMV [7]. Women with primary CMV infection in the first trimester, which can be documented through IgG seroconversion, or detection of positive IgM and IgG antibodies associated with low IgG avidity, have a risk of vertical transmission of almost 30% [8]. Furthermore, infection in early pregnancy is associated with more severe neurodevelopmental outcomes in infected newborns [9].

Serological screening for CMV infection in pregnancy is controversial. Some experts recommend serological screening, given that only 40% of foetuses with congenital CMV infection (cCMV) will show ultrasound anomalies [9]. Most women want to be screened for CMV infection in early pregnancy, once informed about its risks [10]. In addition, high‐dose valacyclovir, an oral antiviral medication, seems to be safe and effective for preventing transmission to the foetus [11].

Previous studies in the United States and France have shown that universal prenatal CMV screening could be cost‐effective only under certain circumstances [12, 13, 14, 15, 16, 17]. However, no such study has been carried out in Canada, where CMV seroprevalence is lower, between 23% and 40% [18] and healthcare costs are different. Thus, we sought to compare the cost‐effectiveness of universal first‐trimester screening for primary CMV infection to targeted serological screening and no serological screening among pregnant women in Canada.

## 2 Methods

We developed a decision‐analytic model tree to compare three strategies for prenatal screening among a theoretical cohort of 80 000 pregnant women in Quebec, Canada, which roughly corresponds to the number of annual pregnancies in the province. These strategies were as follows: 1) Universal first‐trimester serological screening for primary CMV infection; 2) first‐trimester serological screening offered only to women most at high risk, that is, childcare workers and mothers of children under 3 years old; or 3) no serological screening, with CMV testing triggered by abnormalities on foetal morphological ultrasound. The detailed algorithm of these strategies is included in the appendix.

### 2.1 Model Assumptions

In the modelling of Strategy 1, we assumed that universal serological screening was performed only once between 11 and 13 weeks of gestation to detect primary CMV infection in the first trimester of pregnancy in all pregnant women presenting to care. We considered that this screening involved IgG and IgM serology and, if both were positive, IgG avidity. If the IgM was positive in isolation, this strategy required repeating IgG and IgM serology 2 to 3 weeks later. We assumed that valacyclovir 8 g/day was offered to women with serological evidence of primary CMV infection [11, 19]. In this situation, an amniocentesis was offered to perform a CMV polymerase chain reaction (PCR) on the amniotic fluid 8 weeks after the estimated time of primary infection. Valacyclovir was discontinued at the time of the amniocentesis. If amniocentesis was declined, valacyclovir would be continued until delivery. In this strategy, serological screening was not offered to women presenting to care after the first trimester, for whom screening was based on ultrasound only. Ultrasound‐based screening was also provided to all women according to the current routine standard of care.

In the modelling of Strategy 2, we considered that multiparous women and childcare workers were most at risk of developing a CMV infection; first‐trimester CMV serological screening similar to Strategy 1 was proposed only in this group.

In the modelling of Strategy 3, no serological screening was included. However, an amniocentesis to assess foetal cCMV status was offered for cases with abnormal prenatal ultrasound findings [9].

In all three strategies, we assumed that neonatal testing for cCMV was performed in case of identification of CMV primary infection during pregnancy, a positive PCR on the amniotic fluid or in case of symptoms of cCMV identified during pregnancy or at birth. This includes cCMV related to primary and nonprimary infection during pregnancy. Neonates with symptomatic cCMV were treated and followed up according to recommended paediatric care.

To complete our models, some assumptions were made. First, our model of Strategy 1 excluded the possibility of pregnancy termination after positive serological screening without a further evaluation of the status of the foetus for cCMV through amniocentesis, according to current practice in Quebec. Lastly, because there is no gestational age limitation for pregnancy termination in Quebec, we assumed that the choice to terminate the pregnancy could be made at any time during pregnancy follow‐up if there were new prenatal findings.

### 2.2 Probabilities

To obtain base case probability point estimates and confidence intervals, we conducted a focused literature review of articles in English or French in PubMed and Google Scholar on primary maternal CMV infection, serological and ultrasound screening, prenatal diagnosis, maternal–foetal transmission of CMV, medical termination of pregnancy (TOP) and the prognosis of cCMV. We prioritised data from Canada, and then those with better internal and external validity. If there was no superior study in terms of internal or external validity, we used the average of the available data (Table 1). We assumed the risk of cCMV after maternal periconceptional or first‐trimester primary infection to be 28.9% and a reduction of the risk to 11% with valacyclovir [8, 11].

**TABLE 1:** probability estimates.

	Base case (95% confidence interval)	References
First‐trimester screening	94.9% (94.8%–95%)	[20]
Positive serological screening	0.2% (0.1%–0.4%)	[21]
Patients accepting secondary prevention (valacyclovir)	98.2% (90.5%–99.7%)	[22]
Patients accepting amniocentesis	67% (60%–100%)	[23, 24]
Pregnancy loss after amniocentesis	0.3% (0.11%–0.49%)	[25]
Positive amniotic fluid CMV PCR	28.9% (27.3% 30.6%)	[8]
Positive amniotic fluid CMV PCR (with valacyclovir)	11% (6%–18%)	[8, 11]
Termination of pregnancy after positive CMV PCR	20.63% (17.1%–24.7%)	[26, 27, 28, 29, 30]
Normal anatomic ultrasound if positive CMV PCR	45.3% (43.4%–47.2%)	[31]
No ultrasonographic changes in follow‐up	95.6% (93.5%–97.1%)	[31]
Positive amniotic fluid CMV PCR and normal ultrasound		
Liveborn	99.26% (98.4%‐ 99.7%)	[31]
Neurodevelopmental impairment	3.1% (1.6%–5.1%)	[31]
Neurosensorial disorders	6.5% (5.6%–7.5%)	[31]
Positive amniotic fluid CMV PCR and abnormal ultrasound		
Termination of pregnancy	29.4% (21.2%–39.3%)	[28, 32, 33]
Perinatal death	0.6% (0.5%‐ 0.7%)	[34]
Neurodevelopmental impairment	17.9%	[31]
Neurosensorial disorders	11.5%	[31]
Negative predictive value of CMV amniotic fluid PCR with valacyclovir	94.96% (91.8%–97.2%)	[11]
Signs of cCMV on routine ultrasound	4.05% (3.65%–4.45%)	[35]
Amniocentesis following abnormal routine ultrasound	24.69% (23.8%–25.6%)	[36, 37]
Pregnancy loss rates after mid‐trimester amniocentesis	0.06% (0%–0.2%)	[38]
Positive amniotic fluid CMV PCR in cases with ultrasound signs of cCMV infection	8% (5.3%–11.8%)	[39]
High‐risk women	53.5% (53.3%–53.7%)	[40]
Positive serological screening in high‐risk women	0.32%	[21, 41]

Abbreviations: CMV, cytomegalovirus; PCR, polymerase chain reaction.

### 2.3 Costs

We established cost estimates based on 2023 local hospital and provincial insurance data from Quebec [42, 43, 44, 45, 46] (Table 2). These include the costs of serological screening, initial and follow‐up ultrasound, amniocentesis, pregnancy loss, TOP, delivery, neonatal CMV testing, symptomatic neonatal cCMV, perinatal death, mild disability and severe disability with a lifetime horizon. The cost of long‐term care included only medical expenses; loss of productivity due to cCMV was not taken into account as analyses were conducted from a healthcare sector perspective. Costs are expressed in Canadian dollars for 2023.

**TABLE 2:** Deterministic cost‐utility analysis.

		Cost per pregnant woman (95% confidence interval)	Incremental cost economised per pregnant woman*	QALY lost per dyad of pregnant woman and child	Incremental QALY lost per dyad of pregnant woman and child*	Incremental cost‐utility ratio: Incremental cost economised/ Incremental QALY lost
Universal serological screening (Strategy 1)	Lower bound	783 (730–813)	109	−0.42 (−0.29; −0.52)	−0.45	−242
	Mean value	4818 (4221–6780)	2960	−0.42 (−0.32; −0.63)	−0.45	−6577
	Upper bound	9827 (78591–13 235)	6034	−0.42 (−0.36; −0.57)	−0.45	−13 409
High‐risk serological screening (Strategy 2)	Lower bound	838 (773–890)	54	−0.63 (−0.51; −0.77)	−0.24	−225
	Mean value	6196 (4739–7051)	1582	−0.63 (−0.56; −0.79)	−0.24	−6550
	Upper bound	12 806 (9326–14 862)	3055	−0.63 (−0.52; −0.76)	−0.24	−12 729
No screening (Strategy 3)	Lower bound	892 (819–1057)	—	−0.87 (−0.71; −0.99)	—	—
	Mean value	7768 (5587–8426)	—	−0.87 (−0.73; −1.01)	—	—
	Upper bound	15 861 (12267–17 641)	—	−0.87 (−0.67; −0.92)	—	—

*Note*: Costs are in 2023 Canadian dollars.

* Compared to the Strategy 3.

### 2.4 Utilities

To evaluate the health‐related quality of life (HRQoL) of patients, utilities were derived from published literature. Utilities are used in quality‐adjusted life‐year (QALY) studies to provide a value for different health states within a continuum between 0 (death) and 1 (full health) [47]. To target the most important efficacy criteria as well as the elements that modulate the cost‐effectiveness ratio, three patient partners, including two parents of children with cCMV and a mother of a healthy 1‐year‐old child, were involved in all stages of this project, particularly in the choice of primary outcomes for efficacy. We determined six maternal health states and four neonatal health states of importance for our analysis (Appendix S1).

Utilities at each age were drawn from EQ‐5D‐5L studies conducted in the Quebec general population [48]. To consider the utility population norms and thus not overestimate the loss in QALYs, all values described above were weighted by population norms.

### 2.5 Analyses

The analysis was performed from the healthcare sector perspective. Disutilities were summed to calculate the loss in QALYs associated with CMV infection in each strategy over the life course.

The initial analysis compared the three strategies using probabilities, cost and utilities to estimate differences in maternal and neonatal outcomes, total costs and loss of QALYs for each strategy over the life course and the incremental cost‐utility ratio (ICUR). The primary outcome was the incremental cost per QALY lost; the net monetary benefit was calculated by assuming a willingness to pay threshold of 50 000 CAD. Monte Carlo simulation was used to predict the number of times the model's conclusion (the preferable approach) would be chosen again by varying all values across their feasible ranges at random.

We studied how uncertainty affected our results using one‐, two‐ and three‐way sensitivity analyses. These varied one‐, two‐ or three‐variable estimates, respectively, to determine the threshold of valacyclovir efficacy, the threshold of acceptability of amniocentesis and the threshold of the incidence of primary CMV infection at which universal CMV serological screening would be cost‐effective. ICUR, which evaluates the utility of a new medical intervention compared to an existing one, was used to determine whether the costs associated with universal serological screening and high‐risk patient screening are justified by the additional health benefits provided, compared to ultrasound screening.

Secondary analyses included costs of serological screening, amniocentesis and prophylaxis associated with CMV serological screening in Strategies 1 and 2, relative to the ability of each strategy to detect cCMV cases. The cost associated with the prevention of cCMV cases in Strategies 1 and 2 was similarly modelled.

The analyses were performed using TreeAge Pro Healthcare 2022 (TreeAge Software LLC). No ethical approval for this work was required.

## 3 Results

Serologic screening was the most cost‐effective strategy with an economy of 6577 CAD per loss of QALY avoided compared to no screening (Table 2).

**FIGURE 1:**
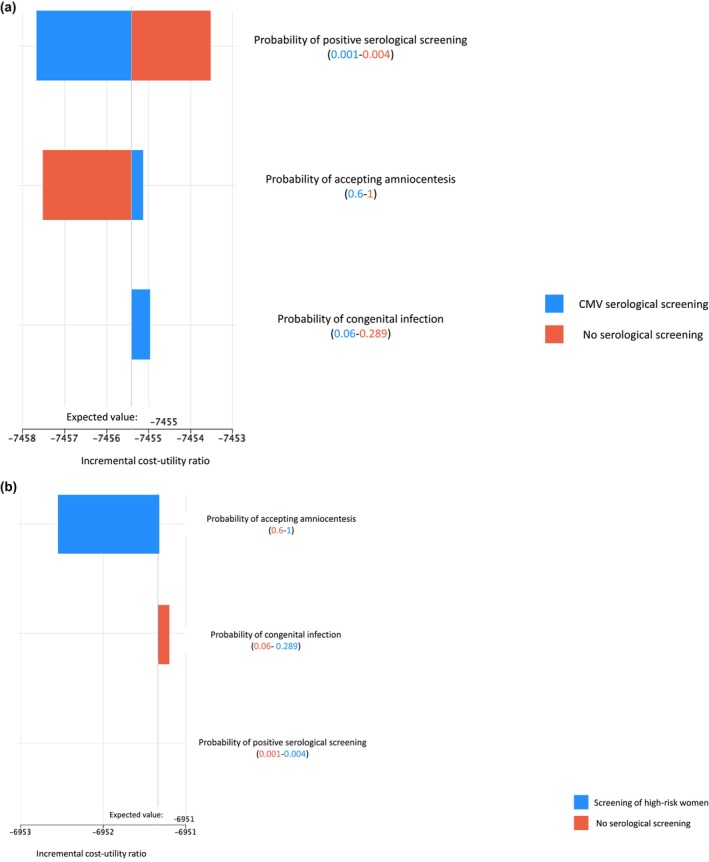
Tornado diagrams. (a) Incremental cost‐utility ratio of universal CMV serological screening versus no serological screening. (b) Incremental cost‐utility ratio of CMV serological screening of high‐risk women versus no serological screening.

**FIGURE 2:**
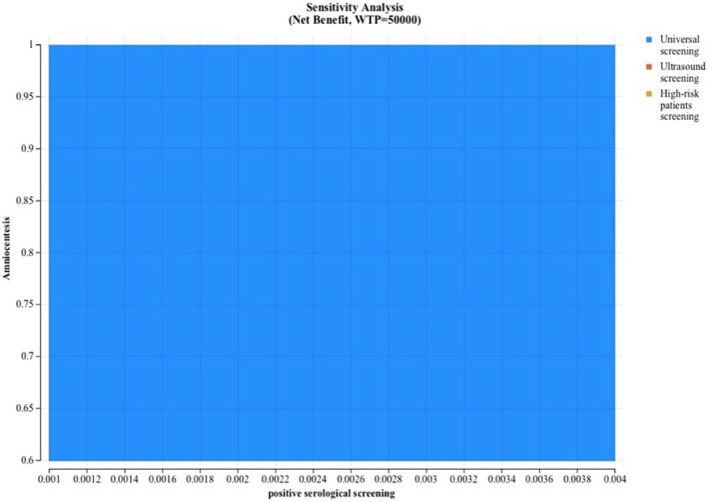
Impact of amniocentesis acceptability and positive serological screening rates on net benefit: Bivariate sensitivity analysis at a 50 000 CAD willingness‐to‐pay threshold.

When conducting one‐way, two‐way and three‐way sensitivity analyses, all strategies remained below the cost‐effectiveness threshold of 50 000 CAD and universal serological screening remained the most cost‐effective strategy (Appendix 3). As an illustration of our sensitivity analyses, a tornado diagram (Figure 1a) demonstrates that the risk of positive serological screening was the variable with the greatest impact on the ICUR of universal CMV serological screening. In contrast, for the ICUR of CMV serological screening in high‐risk women, the probability of accepting amniocentesis had the most significant impact (Figure 1b). However, it should be noted that parameter changes tested in this sensitivity analysis had only a marginal effect on the ICUR. In a bivariate sensitivity analysis assessing how variations in both the acceptability rate of amniocentesis and the percentage of positive serological screenings affect net benefit—using a willingness‐to‐pay threshold of 50 000 CAD—universal screening consistently proves to be the optimal strategy (Figure 2).

**FIGURE 3:**
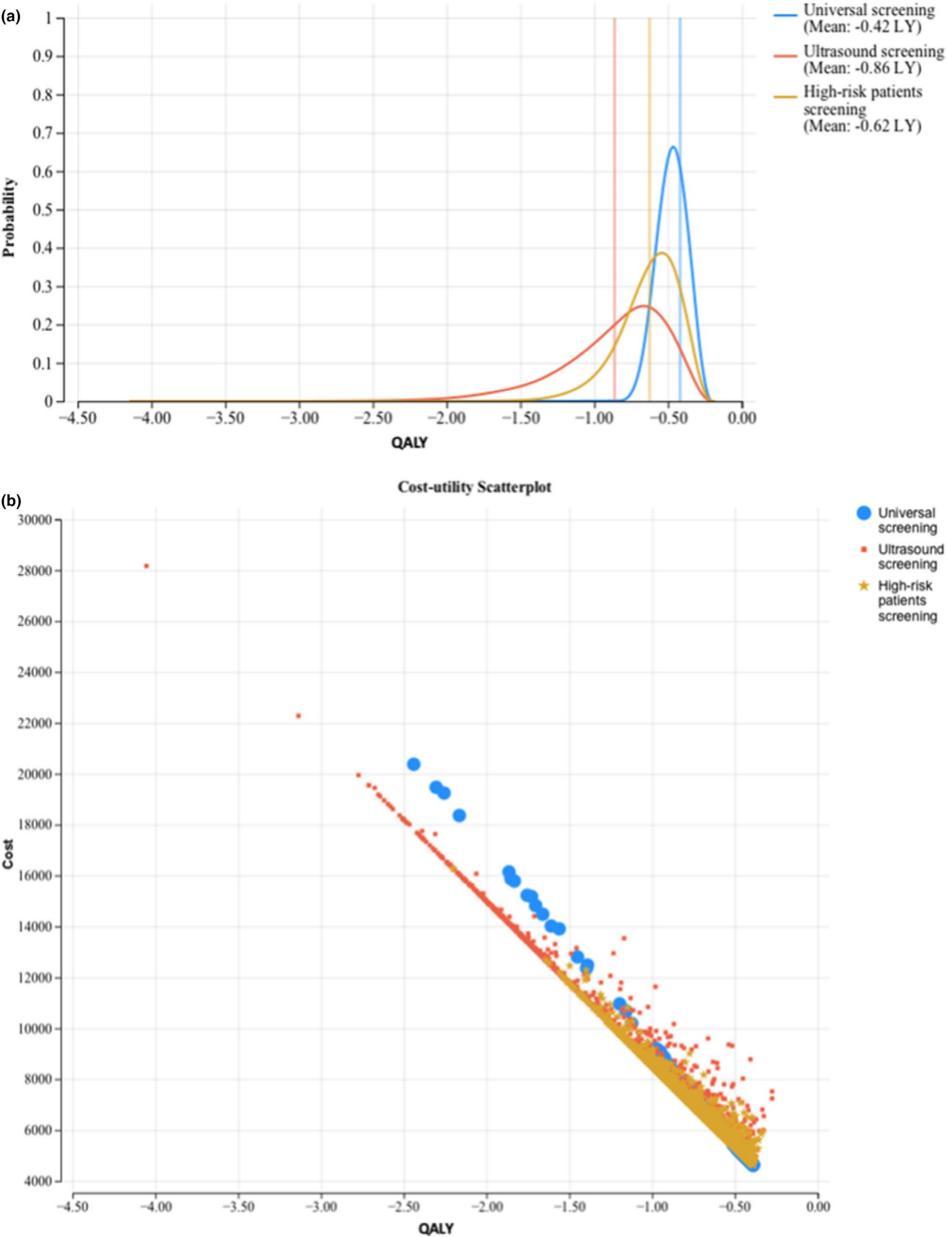
(a) Monte Carlo analysis. LF: Life year. (b) Cost‐utility scatterplot. Dots represent combinations of incremental costs and quality‐adjusted life‐years from 10 000 Monte Carlo iterations for each strategy.

Using a Monte Carlo simulation of 10 000 trials, we conducted a multivariable analysis, simultaneously adjusting probabilities, utilities and costs over their likely ranges. Each strategy was predicted to result in a cost‐utility ratio below 50 000 CAD per QALY lost using minimum, mean and maximum costs. The scatter plot shows that universal serological screening (Strategy 1) is the most cost‐effective option in 96.36% of cases, followed by screening only high‐risk patients (Strategy 2) in 3.51% of cases and ultrasound screening (Strategy 3) in only 0.13% of cases (Figure 3).

In Strategy 1, 75 920 pregnant women would be screened, 152 first‐trimester primary infections would be detected and 46 cases of cCMV would be detected, 29 of which would be prevented by treatment with valacyclovir, with a cost of $94823.52 CAD per case prevented annually.

In Strategy 2, 42 800 pregnant women would be screened, and 27 cases of cCMV would be detected antenatally, 16 of which would be prevented, with a cost of $91949.19 CAD per case prevented annually.

## 4 Discussion

### 4.1 Main Findings

According to our study, a population‐based maternal serological screening is a more cost‐effective approach for CMV screening and prevention than the current routine method based on prenatal morphological ultrasound screening.

### 4.2 Strengths and Limitations

Pregnancy presents a unique challenge for economic evaluations due to its involvement of both the mother and foetus, each with distinct health statuses. A medical intervention that benefits the foetus might pose risks to the mother's health and vice versa. Although QALYs are commonly used in cost‐utility analyses, they may not fully capture maternal or foetal health priorities at different pregnancy stages. Maximising maternal QALY gains could theoretically lead to more pregnancy terminations, resulting in a foetal QALY of 0, as noted by Abel and Quaife [49]. In our study, to mitigate this potential bias, we focused on minimising QALY losses rather than maximising gains. This approach aims to balance maternal and foetal health considerations by reducing potential QALY losses across the study population. It provides a more holistic basis for making informed health policy recommendations, avoiding an overly one‐dimensional view of health outcomes in pregnancy.

Our study marks the first cost‐effectiveness analysis in Canada to tackle the controversial topic of CMV screening in pregnancy. Furthermore, it included modelling of a screening strategy for high‐risk groups (Strategy 2), an aspect not considered in previous cost‐effectiveness analyses. Patient partners were involved in our study design to ensure the selection of the most relevant effectiveness criteria. Finally, we used Monte Carlo simulations to run our model across various cost intervals, considering both best‐case and worst‐case scenarios. The model proved robust across all efficacy criteria.

Our study has several limitations. First, we did not account for the fact that women with a history of CMV‐positive serology from a previous pregnancy would not need serological screening in subsequent pregnancies. This oversight likely overestimates the cost of universal screening, and including this factor would likely strengthen the conclusion that this strategy is the most cost‐effective. Second, due to limited data in the literature and a lack of cost‐specific evidence for Quebec, we made assumptions about the severity of CMV‐related ultrasound anomalies. Specifically, we assumed that congenital cerebral anomalies correspond to severely affected children and congenital extracerebral anomalies to mildly affected children, even though this distinction is not always accurate.

Additionally, our analysis did not consider nonmedical costs, such as those associated with lost productivity or caregiver burden, which could impact the overall economic evaluation. Furthermore, we based our assumptions regarding the efficacy of valacyclovir on a meta‐analysis of one RCT and two prospective cohort studies [19]. However, some experts consider this evidence insufficient; consequently, we accounted for the potential lack of efficacy of valacyclovir in our model.

Lastly, the generalisability of our conclusions may be constrained by the country‐specific unit costs used in the analysis. However, since our analysis is based on comparisons of competing strategies, the cost‐effectiveness conclusions are likely to remain valid in broader contexts. Generalisability of cost‐effectiveness studies is always limited as resource utilisation and monetary values differ substantially across countries and settings. We provide detailed methodology, including the decision tree, key parameters and assumptions, sensitivity analyses and Monte Carlo simulations, which can be adapted to other countries. Our model is particularly relevant for countries with low laboratory resources as we included only one CMV serological screening at the end of the first trimester rather than CMV serology performed early in pregnancy and, in case of a seronegative result, a second serology at 14 weeks of gestation as it was proposed in other cost‐effectiveness studies [14, 50].

### 4.3 Interpretation

In health economics, various methods are available for evaluating cost‐effectiveness, each with unique strengths and limitations. Decision‐analytic modelling, including tools like Markov models and Monte Carlo simulations, is commonly used for projecting long‐term outcomes and assessing intervention cost‐effectiveness. However, its reliability depends heavily on high‐quality input data. Clinical trials and patient data analyses are ideal but not always feasible due to data constraints. Randomised controlled trials are considered the gold standard but are often resource intensive and may not reflect real‐world diversity. Observational studies using real‐world data offer an alternative but are prone to biases. In our study, we utilised decision‐analytic modelling combined with Monte Carlo simulations to manage uncertainties and analyse diverse scenarios, incorporating Canadian data and adjusting for factors like CMV seroprevalence and treatment costs. This approach strengthened the robustness of our conclusions.

Our sensibility analyses demonstrated the superiority of universal serological screening in various circumstances, including the lack of efficacy of valacyclovir for the prevention of cCMV, while this superiority was limited to specific circumstances in other anterior studies conducted in the United States. Thus, the study conducted by Albright et al. showed that the cost‐effectiveness of implementing universal maternal screening for CMV heavily relies on the incidence of primary CMV in pregnancy. In cases where human immunoglobulin (HIG) demonstrates efficacy, universal screening proves to be a cost‐effective approach, particularly in situations with lower primary CMV rates [12]. Similarly, Cahill et al. in another study from the United States reported that universal screening emerged as the preferred and economically efficient strategy only when the efficacy of HIG to reduce the rate of cCMV is above 47% [13]. Fisher et al., on the other hand, demonstrated that the use of universal first‐trimester serologic screening with valacyclovir prophylaxis is not a cost‐effective approach [15]. Because the probabilities, utilities and costs in Canada are quite different from those of the United States, comparing our findings to them is challenging. In addition to that, these studies included CMV HIG for the prevention of cCMV, which is substantially more expensive than valacyclovir but has dubious efficacy [51, 52]. On the contrary, there is evidence demonstrating the efficacy of valacyclovir prophylaxis in preventing vertical CMV transmission [19].

Indeed, other cost‐effectiveness studies which included valacyclovir prophylaxis performed in France [14] and Japan [16] suggested that universal CMV serological screening programmes are cost‐effective compared to programmes based only on prenatal ultrasound, while one US study did not [15]. However, in these studies, CMV seroprevalence was 47%–67%, higher than the seroprevalence in Quebec [20], and costs are also different, limiting the transferability of the results. Indeed, the main argument against CMV serological screening in pregnancy is the fact that serological testing cannot detect CMV reinfections, which are thought to be the leading cause of cCMV in regions of high CMV seroprevalence [53]. However, in Europe and Canada, where CMV seroprevalence is relatively low, half of the cases of cCMV are due to CMV primary infections during pregnancy.

Our study, while sharing some assumptions with the French cost‐effectiveness analysis of CMV screening [50], highlighted key distinctions. In Quebec, the incidence of CMV primary infection is lower (0.002) compared to the French study (0.004). We also incorporated the severity of ultrasound findings, which was not considered in the French model, and calculated the ICUR for each strategy. Additionally, our research included screening for high‐risk groups and compared loss of QALY rather than QALY for each strategy, something previous analyses did not address. By proposing a single first‐trimester screening instead of multiple screenings, we also tailored the approach for countries with fewer resources.

Detecting CMV infections in the first trimester allows for earlier interventions, such as antiviral prophylaxis and targeted foetal monitoring, reducing the risk of severe cCMV and lowering long‐term healthcare costs. In contrast, second‐ or third‐trimester screening delays crucial interventions, increasing the risk of irreversible foetal damage and limiting treatment options. The findings of our study are particularly relevant to countries where late TOP is allowed. In these settings, prenatal screening allows for timely prenatal diagnosis (amniocentesis at least 8 weeks after the infection), assessment of the foetal cerebral anatomy and comprehensive counselling, as recommended in the recently published European guidelines [54]. Screening in settings where late TOP is not allowed would still be relevant for early initiation of valacyclovir and prevention of CMV vertical transmission.

Although CMV is the most common congenital infection and a major contributor to neurodevelopmental impairment, public health interventions for the prevention of cCMV are limited. Education on infection prevention measures has been demonstrated to be moderately beneficial if provided during pregnancy and, as it is usually provided at the first antenatal visit, would not prevent primary infection in the first trimester, which carries a higher risk of severe sequelae associated with cCMV [8].

A population‐based prenatal screening programme that specifically includes CMV serological screening with other prenatal screening tests would allow for the prevention of cCMV, similar to the prenatal screening for HIV, and other sexually and blood‐borne transmitted infections have allowed to decrease the cases of vertical transmission. As most pregnant women present early in prenatal care, this programme would be advantageous for a large proportion of pregnant women. As CMV is more prevalent among disadvantaged populations [18, 21, 55], who are at higher risk of later presentation in prenatal care [56], the implementation of such a programme would need to be accompanied by supporting measures to favour access to the first prenatal visit in the first trimester. The implementation of such screening in Canada would also require the development of counselling tools for patients, training for perinatal care providers and effective laboratory processes in each province.

While our study focuses on prenatal screening for CMV primary infection, neonatal screening has also been demonstrated to be relevant. As described by Letamendia‐Richard et al., universal neonatal screening using saliva PCR tests was not only easy to implement but also well‐accepted by parents, as well as cost‐effective [57]. The value of combining prenatal screening and neonatal screening to reduce severe complications associated with cCMV and ease the burden on healthcare systems remains to be evaluated.

Our study suggests that universal CMV serological screening is superior to ultrasound screening alone and to serological screening restricted to high‐risk women when it comes to cost utility in Canada. The results may eventually contribute to including CMV serological screening in other prenatal screenings.

## Author Contributions

I.B. and T.G.P. have co‐led the project, supervising G.E.H. in all steps, including the conception, planning, data collection, analyses and manuscript writing. S.G., F.K. and C.R. substantially contributed to the conception of the study, interpretation of the results and reviewed the manuscript. M.S and C.M.C. substantially contributed to the data collection, result interpretation and reviewed the manuscript.

## Ethics Statement

The authors have nothing to report.

## Conflicts of Interest

G.E.H., T.G.P., C.R., C.M.C. and M.S. have no conflicts to disclose. F.K. is funded by a ‘Fond de Recherche du Québec – Santé’ salary award; she has received research support from Altona. S.G. has received consultant fees and research funding from Moderna, Merck, VBI, GSK, Meridian Biosciences, Curevo and Seqirus outside of the submitted work. I.B. is funded by a ‘Fond de Recherche du Québec – Santé’ salary award. I.B. has received consultant fees from Moderna and Pfizer, and research support from Altona outside of the submitted work.

## Supporting information


File S1.

Appendix S1.

## Data Availability

Data sharing is not applicable to this article as no new data were created or analyzed in this study.
