# Supplementary figures and images for: Prenatal Screening for CMV Primary Infection: A Cost‐Utility Model
Source: BJOG. 2025 Feb 3;132(6):805–15. doi: 10.1111/1471-0528.18080 (PMC11969920; doi:10.1111/1471-0528.18080)

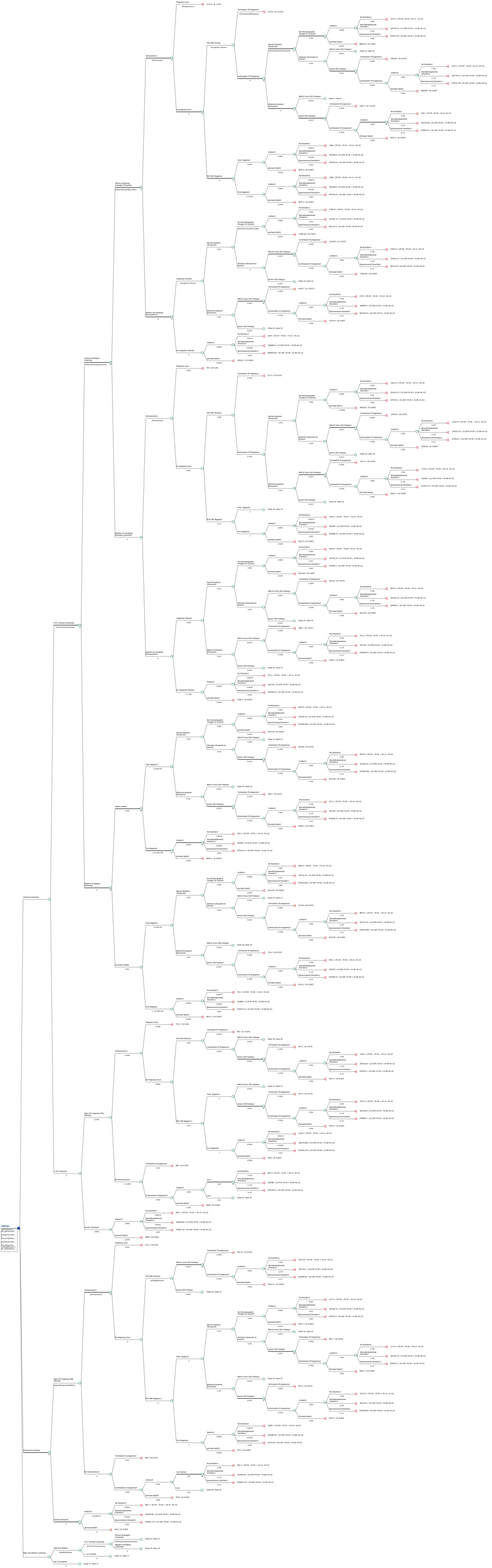

Supplement: Supplementary file 1 — File S1. [file BJO-132-805-s001.png]
