# Supplementary material for: Prenatal Screening for CMV Primary Infection: A Cost‐Utility Model
Source: BJOG. 2025 Feb 3;132(6):805–15. doi: 10.1111/1471-0528.18080 (PMC11969920; doi:10.1111/1471-0528.18080)
Supplement: Supplementary file 2 — Appendix S1. [file BJO-132-805-s002.docx]

**SUPPORTING INFORMATION**

**Appendix 1**

Decision tree diagram for the 3 evaluated strategies

**Appendix 2: Costs**

|  | Costs (in Canadian dollars) | References |
| --- | --- | --- |
| IgM | 17.22 | (21) |
| IgG | 13.27 | (21) |
| IgG Avidity | 79 | (21) |
| Amniocentesis | 105.55 | (22) |
| PCR | 117.45 | (21) |
| Initial ultrasonography | 95 | (22) |
| Follow-up ultrasonography | 44.35 | (22) |
| Valacyclovir 8g daily | 10/day | (23) |
| Creatinine / Alanine aminotransferase | 2.8 | (21) |
| Termination of pregnancy | 470-700 | (22,24) |
| Pregnancy loss | 169- 700 | (22,24) |
| Liveborn | 595.7-825.7 | (22,24) |
| Neurodevelopmental disorders | 2514.3-18441.29 | (25) |
| Neurosensorial disorders | 2440.52-23127.7 | (25) |

Notes: IgM, Immunoglobulin M; IgG, Immunoglobulin G; PCR, polymerase chain reaction

**Appendix 3**

The six maternal health states and four neonatal health states included in the cost-effectiveness analysis:

- The maternal health states included: health after the delivery of a healthy child (utility =1) (55,56), health after delivery of a mildly affected child (utility =0.75, range 0.7-0.8) (55,56), health after delivery of a severely affected child (utility= 0.5, range 0.01-0.9)(55–57), health after pregnancy termination (utility=0.91, range 0.78-0.98) (55,56), health after fetal loss (utility= 0.94, range 0.66-0.99) (55), and health after a perinatal death (utility= 0.92, range 0.6-0.99)(56). Due to a lack of data in the literature, we assumed that a termination of pregnancy, a pregnancy loss, or a perinatal death would reduce maternal quality of life for 1 year. We derived the average maternal age at delivery (31 years) and the average maternal life expectancy 85 years from nationally available data (58,59), to calculate the loss in QALY for mothers with affected children.

The neonatal health states included: unaffected child (utility=1) (55,56), mildly affected child (utility= 0.75, range 0.7-0.8) (55,56), severely affected child (utility= 0.55, range 0.5-0.6)(55–57), and perinatal death/pregnancy termination (utility=0) (56). As for the mother, these data allowed to calculate the loss of utility, and if living it was calculated over the life expectancy of the children. For each healthy infant, an average life expectancy of 82 years was assigned. For mildly affected child, a life expectancy of 65 was assigned, and 20 for severely affected child (57).

**Appendix 4: 1-way sensitivity analysis**

|  | Strategy 1 | | | | Strategy 2 | | | | | Strategy 3 | | | | |
| --- | --- | --- | --- | --- | --- | --- | --- | --- | --- | --- | --- | --- | --- | --- |
|  | Cost (CAD) | Utility (QALY lost) | Cost per QALY lost | Net monetary  benefit | Cost (CAD) | Utility (QALY lost) | Cost per QALY lost | | Net  monetary  benefit | Cost (CAD) | Utility (QALY lost) | Cost per QALY lost | Net  monetary  benefit |  |
| Incidence of primary CMV infection | | | | | | | | | | | | | | |
| 0.1% | 4799 | -0.42 | -11551 | -25773 | 6188 | -0.63 | | -9864 | -37555 | 7745 | -0.87 | -8945 | -51038 |  |
| 0.175% | 4811 | -0.42 | -11538 | -25661 | 6188 | -0.63 | | -9864 | -37555 | 7745 | -0.87 | -8945 | -51038 |  |
| 0.25% | 4823 | -0.42 | -11525 | -25748 | 6188 | -0.63 | | -9864 | -37555 | 7745 | -0.87 | -8945 | -51038 |  |
| 0.325% | 4835 | -0.42 | -11512 | -25836 | 6188 | -0.63 | | -9864 | -37555 | 7745 | -0.87 | -8945 | -51038 |  |
| 0.4% | 4847 | -0.42 | -11499 | -25923 | 6188 | -0.63 | | -9864 | -37555 | 7745 | -0.87 | -8945 | -51038 |  |
| Acceptability of amniocentesis | | | | | | | | | | | | | | |
| 44.3% | 4816 | -0.42 | -11533 | -25692 | 6189 | -0.63 | | -9864 | -37557 | 7745 | -0.87 | -8945 | -51038 |  |
| 46.25% | 4815 | -0.42 | -11533 | -25691 | 6188 | -0.63 | | -9864 | -37556 | 7745 | -0.87 | -8945 | -51038 |  |
| 48.2% | 4815 | -0.42 | -11534 | -25690 | 6188 | -0.63 | | -9864 | -37555 | 7745 | -0.87 | -8945 | -51038 |  |
| 50.15% | 4815 | -0.42 | -11534 | -25689 | 6188 | -0.63 | | -9864 | -37554 | 7745 | -0.87 | -8945 | -51038 |  |
| 52.1% | 4815 | -0.42 | -11534 | -25687 | 6188 | -0.63 | | -9864 | -37553 | 7745 | -0.87 | -8945 | -51038 |  |
| Congenital infection with secondary prophylaxis | | | | | | | | | | | | | |  |
| 6% | 4792 | -0.41 | -11566 | -25510 | 6169 | -0.62 | | -9876 | -37401 | 7745 | -0.87 | -8945 | -51038 |  |
| 11.725% | 4798 | -0.42 | -11558 | -25555 | 6174 | -0.63 | | -9873 | -37439 | 7745 | -0.87 | -8945 | -51038 |  |
| 17.45% | 4804 | -0.42 | -11550 | -25600 | 6178 | -0.63 | | -9870 | -37478 | 7745 | -0.87 | -8945 | -51038 |  |
| 23.175% | 4809 | -0.42 | -11542 | -25645 | 6183 | -0.63 | | -9867 | -37516 | 7745 | -0.87 | -8945 | -51038 |  |
| 28.9% | 4815 | -0.42 | -11534 | -25690 | 6188 | -0.63 | | -9864 | -37555 | 7745 | -0.87 | -8945 | -51038 |  |
